# Staff Perspectives of Implementing a Community‐Based Stroke Recovery Program in Regional Australia: A Qualitative Study

**DOI:** 10.1111/ajr.70247

**Published:** 2026-10-12

**Authors:** Jaclyn Lewis, Marie‐Louise Bird, Liam Johnson, Lauren J. Christie, Christine T. Shiner, Dawn B. Simpson, Katrina Kenah, Heidi Janssen, Kirsti Haracz

**Affiliations:** ^1^ Hunter New England Local Health District Newcastle New South Wales Australia; ^2^ School of Health Sciences, College of Health, Medicine and Wellbeing University of Newcastle Newcastle Australia; ^3^ Institute of Health and Well Being, Federation University Churchill Victoria Australia; ^4^ Department of Physical Therapy University of British Columbia Vancouver Canada; ^5^ School of Health Science University of Tasmania Launceston Tasmania Australia; ^6^ Institute for Musculoskeletal Health, Sydney Local Health District Sydney Australia; ^7^ School of Health Sciences, Faculty of Medicine, Dentistry and Health Sciences University of Melbourne Melbourne Australia; ^8^ Physiotherapy Department Epworth HealthCare Melbourne Australia; ^9^ School of Behavioural and Health Sciences, Faculty of Health Sciences Australian Catholic University Melbourne Australia; ^10^ Allied Health Research Unit St Vincent's Health Network Sydney Darlinghurst New South Wales Australia; ^11^ School of Allied Health, Faculty of Health Sciences Australian Catholic University North Sydney New South Wales Australia; ^12^ Nursing Research Institute St Vincent's Health Network Sydney, St Vincent's Hospital Melbourne, and Australian Catholic University Darlinghurst New South Wales Australia; ^13^ St Vincent's Centre for Applied Medical Research, St Vincent's Hospital Sydney Australia; ^14^ Health Equity Research St Vincent's Health Australia Sydney Australia; ^15^ School of Clinical Medicine, Faculty of Medicine and Health University of New South Wales Sydney Australia; ^16^ Heart and Stroke Program Hunter Medical Research Institute Newcastle Australia; ^17^ Workforce, Innovation, Strategy, Education and Research Unit Monash Health Melbourne Australia

**Keywords:** behaviour change, enriched environment, implementation, staff, stroke

## Abstract

**Objective:**

To describe the experience of non‐government organisation (NGO) staff implementing a community‐based stroke recovery program based on the principles of environmental enrichment.

**Design:**

A qualitative descriptive study to capture staff experiences. Semi‐structured interviews with staff were analysed using reflexive thematic analysis.

**Setting:**

A community‐based NGO service in regional New South Wales, Australia.

**Participants:**

Eight staff members employed in a regional NGO and involved in the implementation of the program, including a program coordinator, a physiotherapist, allied health assistants, creative artists and managers.

**Results:**

Four themes were identified: (1) We've got a great space here—highlighted the importance of the physical environment, access and location; (2) There's an order to it—described the program's strong foundations formed through training, defined processes and support; (3) We make it work—demonstrated the flexibility and adaptability of staff in responding to individual needs and overcoming challenges and (4) Enriching lives—captured the sense of community, positivity and recovery outcomes. Staff experiences aligned closely with self‐determination theory, reflecting feelings of capability, confidence and connection.

**Conclusion:**

Staff in a regional NGO felt well‐supported and capable of delivering a community‐based stroke program. This demonstrates the potential role NGO services may play in addressing gaps in stroke recovery care in regional Australia. The methods used in this study can be used to implement this and other similar community‐based programs with NGO services in the future.

## Abstract (highlights)

**What is Already Known on This Subject?:**

• Community‐based stroke programs can assist recovery after hospital discharge, but access to such programs is limited in rural and regional areas of Australia.
• Implementation of multi‐component evidence‐based programs in the community to address this limitation, especially within a regional non‐government organisation (NGO) setting, is under‐explored.

**What Does This Study Add?:**

• Actively engaging non‐government organisation (NGO) staff in implementation planning helps staff feel well supported to deliver a multi‐component stroke recovery program.
• Implementation strategies that support staff competence, autonomy and connectedness, enhance confidence and capability to deliver a multi‐component stroke recovery program.
• NGO staff can deliver a multi‐component community‐based stroke recovery program in a regional community.

## 1 Introduction

Stroke is a leading cause of long‐term disability, with many survivors requiring ongoing community‐based rehabilitation to support their recovery [1]. Although in‐hospital stroke rehabilitation services are well‐established, access to ongoing programs after hospital discharge remains limited [2], particularly in regional and rural areas [3]. Workforce shortages [4], geographic isolation [5] and other systemic challenges contribute to these inequities, leaving stroke survivors in these settings with minimal opportunities to engage in interventions crucial for long‐term recovery [6]. To address this service gap, rather than relying solely on traditional healthcare services, innovative models of care that leverage community resources and infrastructure [7] are needed.

The Exercising, Socialising and Thinking: Environmental Enrichment Model (ESTEEM) Program was developed in collaboration with a community‐based non‐government organisation (NGO) to enhance access to community‐based stroke rehabilitation and recovery supports. Grounded in the principles of environmental enrichment, which describes multi‐modal stimulation achieved through participation in a combination of physical, social and cognitive activities [8], the program provides opportunities for stroke survivors to engage in novel and stimulating exercise, social and thinking activities. Use of environmental enrichment in animal models with stroke contributes to significant improvements in neuroplasticity and subsequent sensorimotor function [9]. Evidence emerging from the application of environmental enrichment with stroke survivors in hospital and community settings is associated with increased activity levels [10], improved mood [11] and enhanced self‐perceived recovery [12]. The ESTEEM Program, co‐designed with stroke survivors, carers, healthcare clinicians and service providers, is under evaluation in a randomised wait‐list controlled trial (ACTRN 12625000270415). The program is delivered twice weekly for 10 weeks. Each group session incorporates 30 min of group exercise, a 30‐min social morning tea and 90 min of creative thinking activities (i.e., visual arts or movement to music) [13].

NGOs play a vital role in addressing health service gaps [14], yet their involvement remains under‐utilised [15]. Their community‐based focus and operational flexibility position them uniquely to deliver tailored, evidence‐based interventions where formal public health services may be limited [15]. NGO‐led programs have proven effective in increasing access to healthcare services in the community setting across different contexts [16, 17]. However, despite evidence of program effectiveness, limited attention has been paid to understanding the experiences of NGO staff delivering these community‐based programs [18]. This represents a significant gap, given their essential role in implementing and scaling community‐based interventions in underserved regions.

Successful implementation of novel interventions in new settings relies heavily on the engagement of those delivering the interventions. Knowledge of staff perspectives is critical, as their capability, opportunity and motivation directly influence program implementation success [19]. Behaviour change tools such as the Capability Opportunity Motivation—Behaviour (COM‐B) model [19] and the Theoretical Domains Framework (TDF) [20] offer structured, theory‐based approaches to examining implementation processes and have been successfully applied with healthcare professionals working with stroke survivors. These frameworks are typically used prospectively to identify barriers and facilitators or retrospectively to inform the design of interventions [21, 22], although their application beyond acute care settings or in the design of interventions has been limited [23]. The iterative application of these frameworks to inform implementation and capture staff experiences is a unique approach that supports responsiveness to local contexts while maintaining theoretical rigour in understanding implementation mechanisms.

In this study, we aimed to describe the experiences of NGO staff involved in the implementation of the ESTEEM Program in regional Australia. The outcomes of this work will inform refinement of implementation strategies and support implementation of the ESTEEM Program with additional NGO services in the future.

## 2 Methods

### 2.1 Study Design

We used a qualitative descriptive approach, which aligned with our aim and enabled the firsthand perspectives of staff to be the focus. This design supports a comprehensive account of reported experiences from the viewpoint of staff and is widely used in healthcare research [24]. The Consolidated Criteria for Reporting Qualitative Research (COREQ) Checklist [25] was used to guide reporting. Ethics approval was from Hunter New England Local Health District Human Research Ethics Committee (2020/ETH01723).

### 2.2 Sampling and Recruitment

A purposive sampling strategy was used. All staff employed by the participating NGO and involved in the implementation of the ESTEEM Program were invited to participate. Recruitment occurred via email. Participation was voluntary and verbal consent was obtained at the start of each interview.

### 2.3 Context

This study was embedded within the implementation phase of the multi‐site ESTEEM After Stroke Research Trial (ACTRN12625000270415). Prior to this study, systematic theory‐driven processes using the COM‐B model and the TDF were used iteratively to understand local context and map barriers, facilitators and implementation considerations (Figure 1), directly influencing the outcomes. This work will be presented separately.

**FIGURE 1:**
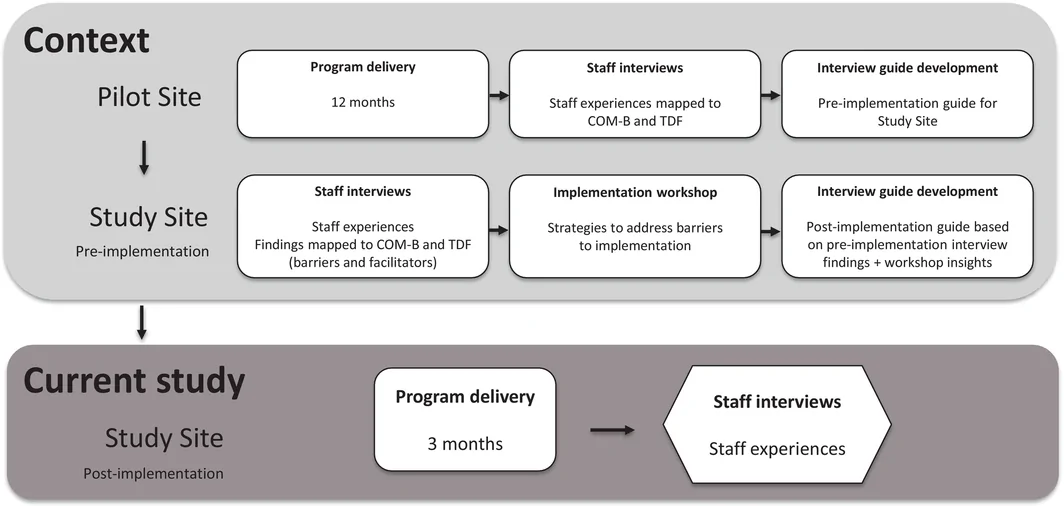
Iterative process. Capability, Opportunity, Motivation—Behaviour (COM‐B) and Theoretical Domains Framework (TDF).

### 2.4 Data Collection

Semi‐structured interviews were used to collect qualitative data, which allowed for focused yet flexible discussions. This approach enabled staff to share detailed insights, facilitated follow‐up questions based on responses and provided opportunities for relevant data to be explored.

#### 2.4.1 Interview Guide

Insights and data gathered from thematic analysis of the pre‐implementation interviews and the implementation workshop conducted with the Study Site staff were used to develop the post‐implementation interview guide. This interview guide was trialled for use with a member of the research team familiar with the study context but not involved in the development of the interview guide. Based on this feedback, minor refinements were made to the wording of questions to improve clarity and flow. The final interview guide contained seven questions (Table 1).

**TABLE 1:** Post‐implementation interview guide.

How well prepared were you to implement the program?
2 How did your physical environment or setting impact on the delivery of the program?
3 How did the people around you and [the Study Site NGO] impact on the delivery of the program?
4 How did service processes impact on the delivery of the program?
5 What were your impressions of how the ESTEEM sessions ran?
6 What changes did you or others make to the program as it progressed to help it work better in your centre?
7 What benefits have occurred as a result of ESTEEM running at [the Study Site NGO]?

#### 2.4.2 Procedure

Individual interviews were conducted online via Zoom in November and December 2024, 3–5 months after program implementation, by the first author (J.L.), a female Physiotherapist working as a stroke care coordinator in regional NSW and a PhD candidate at the University of Newcastle. J.L. had no clinical working relationship with the study participants. Interview training and supervision of J.L. was provided by K.H., a female Occupational Therapist with a PhD and researcher with expertise in qualitative research methods. All interviews were audio and video recorded, transcribed using Microsoft Word's built‐in transcription feature, checked for accuracy and de‐identified through the assignment of pseudonyms by J.L. prior to analysis.

### 2.5 Data Analysis

Interview transcriptions were imported into NVivo 14 [26] and analysed using reflexive thematic analysis (RTA). This interpretive, researcher‐driven approach [27] was selected for its alignment with the study's aim of understanding the participants' experiences and is well‐suited for exploring under‐researched areas [24].

JL led the analysis with guidance and regular feedback from KH, following Braun and Clarke's six phases of RTA [28]: (1) data familiarisation, (2) data coding, (3) initial theme generation, (4) theme generation and review, (5) theme refining, defining and naming and (6) writing up. In Phase 1, J.L. familiarised herself with the data by checking transcripts and reviewing recordings. In Phase 2, J.L. developed initial codes with oversight from K.H. Phases 2–3 involved initial coding, journaling observations and generating preliminary themes with K.H. In Phase 4, codes and themes were revised by J.L. and K.H., then presented to the broader research team for feedback. Phase 5 involved naming themes and drafting descriptive accounts by JL with oversight from KH. In Phase 6, JL produced a report detailing themes, subthemes and exemplar quotes.

### 2.6 Rigour

Rigour was addressed through the principles of credibility, transferability, dependability and confirmability [29]. Credibility was enhanced through prolonged engagement across multiple timepoints, allowing JL to develop familiarity with the study context. Transferability was supported by providing rich descriptions of the study context, setting and implementation processes. Dependability was established by maintaining systematic research processes with clear audit trails, including regular meetings between JL and KH during analysis to discuss interpretations and ensure consistency. Confirmability was strengthened through peer debriefing, inclusion of participants' quotes linking findings to the data and reflexivity [30], with JL maintaining a reflexive journal to document assumptions, reactions and insights.

## 3 Results

All invited staff participated in the interviews that lasted 40–60 min each. Interviews were conducted with eight staff representing a range of clinical, creative, coordination and managerial roles within the program. Staff roles are described in Table 2. Seven were female and four had prior experience delivering the program at the Pilot Site.

**TABLE 2:** Staff roles.

Position title	Description of role
Program coordinator	Overall coordinator of the program and stroke survivor journey. Coordinated stroke survivor intake, orientation to and overall journey through the program. Main contact for stroke survivors and staff, liaised with transport and supported overall team as needed.
Physiotherapist	Conducted physical intake assessment, individualised exercise programs and led group exercise sessions.
Allied health assistant	Supported stroke survivor participation in all program elements. Supported physiotherapist and creative artists. Supported social morning tea.
Creative artist	Led creative thinking sessions. Supported social morning tea.
NGO manager	Oversight of the staff. Program oversight within the NGO.

Four themes were identified, capturing staff experiences of implementing the ESTEEM Program. These themes reflected the importance of the physical environment and location; the value of well‐defined processes and preparation; the flexibility and adaptability required in day‐to‐day delivery of the program; and the perceived benefits of the program. Themes and subthemes are presented in Table 3.

**TABLE 3:** Themes and subthemes.

We've got a great space here	All in one space
	Easy set up and transition
	Great access
	Amazing location
There's an order to it	We were well‐prepared
	Having a clear process
	Getting people to the program
	Being supported
We make it work	Adapting to individual needs
	Doing what is needed
	There's another way
Enriching lives	Sense of community
	Positive and helpful
	Supporting recovery

### 3.1 Theme 1: We've Got a Great Space Here

The physical environment was perceived as well‐suited to program delivery, with staff highlighting benefits of the open layout, accessibility and proximity to the natural environment.

#### 3.1.1 All in One Space

Delivering the program in one large open space was seen to be supporting group cohesion and observation. Quinn stated ‘everyone feels like they're in a big group still and not broken away into two separate groups’. Monitoring the stroke survivors at all times was perceived as beneficial, ‘if I'm sitting typing my notes, I can see what's happening, and I can hear if somebody is more tired than usual or they've got a niggle’ (Avery).

#### 3.1.2 Easy Set Up and Transition

The layout supported efficient program set up and smooth transition between activities, ‘we don't have to be setting up in between activities, so people are not standing there waiting for us to do stuff… It's all done’ (Riley). Staff found it easy to move around both during and in between activities, ‘it's not crammed in any way’ (Asher). Staff found the convenient location of the equipment storage room next to the exercise area assisted set up.

#### 3.1.3 Great Access

Internal and external access were described as well suited to the program and needs of the stroke survivors. Parking for staff and stroke survivors was perceived as appropriate and convenient, ‘there's lots of car parking, it's free car parking. I mean, what more do you want? (laughs)’ (Remy). Access into the building was easy, ‘we've got a driveway right next to it, nice open doors, flat, no stairs’ (Charlie). The bathroom and kitchen facilities were described as conveniently located. However, not all staff had a key to the building, ‘sometimes we have to wait’ (Asher).

#### 3.1.4 Amazing Location

Proximity to the natural environment was viewed as positive for staff and stroke survivors. Asher said, ‘it's a beautiful walk out near the bush … they [stroke survivors] quite like that nature aspect after the exercise session … it's fantastic.’ Being located on the grounds of a public health site facilitated access to resources and promotion opportunities to the local rehabilitation service, ‘they've [other staff] seen it, they're [other staff] starting to refer which is great’ (Riley).

### 3.2 Theme 2: There's an Order to It

There were strong foundations for establishing and implementing the ESTEEM Program, with staff reporting feeling well‐prepared and supported through defined processes and resources, and an ordered way for people to access the program.

#### 3.2.1 We Were Well‐Prepared

Prior experience of working with stroke survivors and the workshop supported feeling well‐prepared. Some staff brought prior experience of delivering the program at the Pilot Site, which was perceived to be particularly beneficial, with Jamie reporting ‘we were really lucky… because the knowledge that they brought with them, I think really helped support our staff’. The workshop training was described as informative by Charlie ‘we were learning a lot about the benefits.’ Training was described as ‘very thorough’ (Asher) and supporting a good understanding of the need to maintain trial intervention fidelity, ‘you gotta stick to the program’ (Remy). To further support this understanding, early exposure to the program, via video or visiting another site was suggested, ‘I still didn't really get an idea, couldn't visualise what the program was … that would have been helpful’ (Charlie).

#### 3.2.2 Having a Clear Process

Well‐defined processes and extensive resources for delivering the program were valued by staff. However, additional instruction in their use could be beneficial, ‘I could have done with a bit more information around the documents’ (Charlie). During the workshop, staff discussed implementing a regular team ‘huddle’, a brief meeting held each morning of the program. This huddle was perceived as essential for team communication and problem solving, ‘we discuss things, we nut it out’ (Quinn) and it's ‘where we get most of the information’ (Riley) about the stroke survivors.

#### 3.2.3 Getting People to the Program

While there were clear processes for program intake, staff reported this was often a time consuming and frustrating task. The intake process was ‘quite long … it does take up a lot of time’ (Emerson) and incomplete referral forms contributed to the frustration. Furthermore, intake phone calls were described by Charlie as ‘quite challenging … quite confronting’ when the person did not meet the criteria for the program or were having difficulties at home. Getting medical clearances from General Practitioners was described as laborious, ‘we ring and ring … it slows it right down’ (Riley). In contrast, after commencing the program, getting stroke survivors to the program using the NGO transport was described as a well‐coordinated, easy and smooth process. Transport arrived ‘at the right hours and the right times … it's been really good’ (Avery).

#### 3.2.4 Being Supported

Staff described feeling supported by the research team. Interactions were perceived to be a positive experience, ‘they were happy… we were happy’ (Remy). The research team was described as available, responsive and ‘great to work with’ (Jamie). In contrast, some staff described feeling there was a lack of timely communication from the NGO, ‘we just sort of needed a little bit more information earlier’ (Quinn).

### 3.3 Theme 3. We Make It Work

This theme centred around the idea that the staff adapted their practice and could be flexible in program delivery as needed.

#### 3.3.1 Adapting to Individual Needs

Staff described tailoring aspects of the program to suit the individual needs of stroke survivors and their carers. This included modifying activities and/or providing additional assistance, ‘you might need to support someone with higher needs’ (Charlie). During ‘movement to music’, different ways of moving was encouraged, ‘I always say to them, it's not wrong, it's just different’ (Avery). Staff described prioritising individual support when needed, while maintaining group engagement as part of their role. Sometimes additional equipment was sourced from the public health site where the program was delivered to enhance safety and participation, ‘we were able to get the right equipment so they could still attend’ (Emerson).

#### 3.3.2 Doing What Is Needed

A willingness and ability to be flexible and do what was needed to deliver the program was expressed, despite staffing changes or unexpected issues with stroke survivors. Emerson said, ‘everyone helps out regardless of what their role is’. When colleagues were absent, roles were adapted ‘so that the sessions still run as planned’ (Jamie) or the program was adapted, for example, by having the whole group engaged in one creative activity instead of being divided between two. Providing emotional support to stroke survivors was described.

#### 3.3.3 There's Another Way

Staff found alternative ways of working when presented with resource or environmental challenges. However, these often resulted in increased workload, time and effort. A slow internet connection led to mobile phones being used for internet connection. Accessing a photocopier was challenging, resulting in staff juggling time and program demands during activities and completing tasks outside of their allocated work time, ‘it's not easy… it's time consuming’ (Quinn).

### 3.4 Theme 4. Enriching Lives

The program was perceived to be enriching lives through a strong sense of community, a culture of positivity and helpfulness and by supporting recovery after stroke.

#### 3.4.1 Sense of Community

Strong relationships were described. Stroke survivors were ‘engaged with one another’ (Charlie) and showed care for one another. Efforts were made by the stroke survivors to maintain relationships after program completion, ‘there's a lot of people who've shared contacts which is really nice’ (Asher). Staff also described strong relationships among their colleagues, ‘we are like a little family, which is lovely’ (Asher). Relationships between NGO transport workers and stroke survivors were also reported as important, ‘that's where they're building relationships, talking in the car’ (Charlie).

#### 3.4.2 Positive and Helpful

A positive, supportive and enriching culture was described for staff and stroke survivors. Staff worked collaboratively, ‘we're the dream team’ (Quinn). Flexible and cooperative ways of working was felt to enhance job satisfaction and enjoyment of staff. The emotional impact of receiving feedback and appreciation from stroke survivors was evident, ‘it's a bit of enrichment in our life as well to be able to see how happy people are leaving and how grateful they are to be in the program’ (Charlie). Helping stroke survivors and carers by sharing information and providing support was demonstrated, ‘they can ask questions, I'm always happy to help’ (Remy).

#### 3.4.3 Supporting Recovery

Staff observed a range of psychological and physical improvements among the stroke survivors. Increased confidence was highlighted as a noticeable benefit, ‘I think they all, and I mean all of them, are getting more confidence out of it’ (Remy) as well as enhanced emotional well‐being. These psychological improvements were seen as contributing positively to improved quality of life of the stroke survivors. Improvements in strength and mobility were also observed, ‘you see people coming in with crutches and walking frames and they walk out with no crutches’ (Remy).

## 4 Discussion

This study provides valuable insights into the experiences of NGO staff implementing the ESTEEM Program, a community‐based stroke recovery intervention, in a regional community in NSW, Australia. Behaviour change frameworks were iteratively applied to inform delivery strategies and capture staff experiences. There are four overarching themes from this study: (i) we've got a great space, (ii) there's an order to it, (iii) we make it work and (iv) enriching lives. Across these themes, staff expressed a sense of capability, confidence and connection.

These three elements align with the core components of self‐determination theory (SDT); a theory which explains that optimal functioning and wellness depend on the satisfaction of three core psychological areas: competence, autonomy and relatedness [31]. When these psychological needs are met, individuals are more likely to experience internalised motivation, embracing personally endorsed values rather than responding to external pressures. Internalised motivation supports sustained engagement and sustainable practice change [32]. In workplace contexts, SDT‐informed strategies such as supporting autonomy by giving background knowledge, offering choice and allowing employees to express opinions are associated with greater employee engagement, satisfaction and enhanced internalised motivation [33]. The implementation approach in this study, including tailored training, collaborative decision making and relationship building, may have created conditions that promote internalised motivation among NGO staff.

In this study, staff capability aligned with the SDT's construct of competence, defined as feeling effective and capable to perform tasks in one's role [34]. Competence is achieved when individuals perceive they have the necessary skills and resources to meet challenges successfully. Strategies that supported competence included tailored training, access to resources and ongoing support from colleagues and the research team. Building staff capability in healthcare improves patient outcomes, organisational work practices and staff engagement [35]. In this context, strengthening competence may enhance stroke survivor outcomes in regional communities, increase NGO's organisational capacity to deliver evidence‐based programs and foster staff motivation in delivering specialised interventions like the ESTEEM Program.

Autonomy, as defined by SDT, refers to the need to experience choice and control over one's actions [34]. In this study, autonomy was demonstrated through staff confidence in making contextually appropriate adaptations and problem‐solving collaboratively during program delivery. Staff were confident in the expertise of colleagues to maintain program structure while adapting to local needs, a reflection of real‐world implementation practices where flexibility is necessary [36]. Supporting autonomy has been identified as a key capacity‐building strategy and critical for scaling public health interventions [37]. In regional NGO settings, fostering autonomy enables staff to feel ownership of programs and respond to local needs while maintaining program fidelity, which may assist sustainability.

Relatedness, as defined by SDT, represents the need to feel a sense of attachment and belonging within a group [34]. In this study, staff described connection through professional and personal fulfilment, reflecting strong relationships and shared purpose in delivering meaningful work. These findings align with previous research showing that trusting relationships foster meaningful professional engagement across multidisciplinary stroke care teams [38]. For NGOs, cultivating relatedness among staff may strengthen team cohesion, reflecting the supportive and positive culture created in ESTEEM Program implementation.

Foundational to staff experience was the physical context in which the program was implemented. The importance of the physical environment in a community stroke recovery program, as captured in Theme 1 ‘We've got a great space here’, is consistent with stakeholder perspectives on environmental factors in stroke rehabilitation [39]. Stakeholders, including healthcare professionals, managers and stroke survivors, identified accessible outdoor environments, proximity to other services, safe and appropriate access, parking facilities and spaces that support social participation, as important contributors to stroke recovery in the community. Together, these findings suggest that delivering community stroke programs in physically accessible, logistically convenient settings with access to natural surroundings may support the experiences of both stroke survivors and staff.

This study addresses a gap in the literature by offering a structured approach to capturing the perspectives of an under‐researched workforce in NGO‐led community‐based health service delivery [40]. It contributes to understanding across multiple domains including implementation science, NGO workforce experiences and community‐based stroke recovery programs. The conduct and evaluation of the implementation of a new stroke recovery program with a regional NGO service provider in the community setting, extends existing known models of community‐based stroke care and highlights practical strategies to employ in the future with NGO staff delivering community‐based programs beyond stroke. The findings reinforce the important potential role NGO services may play in addressing the persistent service gaps in community healthcare, particularly in regional areas. Lastly, the results from this study may inform implementation of the ESTEEM Program with new additional NGO services, and other providers of stroke recovery care (e.g., health professionals and workers within private and public health care systems).

### 4.1 Strengths and Limitations

This study has several strengths. We used a theory‐informed approach to understanding implementation experiences of health professionals in real‐time. Comprehensive sampling was achieved, with all staff involved in the ESTEEM Program implementation at different levels of the participating NGO, ensuring that the findings reflected a broad understanding of their experiences and organisational perspectives. Additionally, the multi‐disciplinary research team, including stroke researchers and healthcare professionals, enriched the study design and implementation.

The study also had limitations. The study was conducted within a single regional NGO, which may limit the transferability of findings to other organisational or geographical contexts. Furthermore, while the pragmatic nature of the implementation may facilitate timely translation of evidence into practice, it may also influence findings in ways that limit their transferability to other contexts.

## 5 Conclusion

This study describes the experiences of NGO staff involved in the implementation of a community‐based stroke recovery program in a regional setting. The alignment between staff experiences and SDT, specifically the development of capability, confidence and connection, suggests that this approach to implementation was supportive for participating staff. This study contributes to implementation science by examining an under‐researched workforce and service context and highlights the potential role NGO services may play in addressing the gap in access to recovery care that many stroke survivors living in regional communities experience.

## Author Contributions

**Jaclyn Lewis:** conceptualisation, data curation, formal analysis, methodology, investigation, project administration, writing – original draft preparation, writing – review and editing. **Marie‐Louise Bird:** conceptualisation, methodology, project administration, supervision, writing – original draft preparation, writing – review and editing. **Liam Johnson:** conceptualisation, writing – review and editing. **Lauren J. Christie:** conceptualisation, methodology, writing – review and editing. **Christine T. Shiner:** conceptualisation, writing – review and editing. **Dawn B. Simpson:** conceptualisation, writing – review and editing. **Katrina Kenah:** writing – review and editing. **Heidi Janssen:** conceptualisation, funding acquisition, methodology, project administration, supervision, writing – original draft preparation, writing – review and editing. **Kirsti Haracz:** conceptualisation, data curation, formal analysis, supervision, writing – original draft, writing – review and editing.

## Funding

This study was funded by the Australian Government Medial Research Future Fund (MRFF 2024071). The funder had no role in the study design, data collection, data analysis, data interpretation or report writing.

## Disclosure

The authors confirm that no generative artificial intelligence tools were used in the writing or preparation of this manuscript.

## Conflicts of Interest

The authors declare no conflicts of interest.

## Data Availability

The data that support the findings of this study are available from the corresponding author upon reasonable request.
